# Fluid-Dynamic Crestal Sinus Floor Elevation in Atrophic Posterior Maxilla Implant Rehabilitation with Hyaluronic Acid: A Prospective Study

**DOI:** 10.3390/ma18102230

**Published:** 2025-05-12

**Authors:** Alessandro Scarano, Roberto Luongo, Ilaria De Filippis, Antonio Scarano, Erda Qorri, Francesco Sforza, Mario Rampino, Calogero Bugea

**Affiliations:** 1 Private Practice, Via Mons A. Semeraro 19, 72021 Francavilla Fontana, BR, Italy; 2 Private Practice, Via Melo da Bari 229, 70122 Bari, BA, Italy; robyluo@tin.it; 3 Private Practice, Via Valle d’itria, 7, 72023 Mesagne, BR, Italy; ilaria.defilippis@icloud.com; 4 Department of Medical, Oral and Biotechnological Sciences, University of Chieti-Pescara, 66100 Chieti, CH, Italy; ascarano@unich.it; 5 Department of Dentistry, Faculty of Medical Sciences, Albanian University, 1001 Tirana, Albania; e.qorri@albanianuniversity.edu.al; 6 Private Practice, Corso Umberto I 115, 72012 Carovigno, BR, Italy; info@studiosforzaromano.it; 7 Private Practice, Via Giordano Bruno 2, 72020 Tochiarolo, BR, Italy; rampino.mario@libero.it; 8 Private Practice, Lungomare G. Galiei 133, 73014 Gallipoli, LE, Italy; calogerobugea@yahoo.it

**Keywords:** hydrodynamic sinus lift, sinus floor augmentation, transcrestal sinus floor elevation, Flusilift, hyaluronic acid, graftless, fluid-dynamic sinus augmentation, crestal approach

## Abstract

Implant–prosthetic rehabilitation of the posterior edentulous maxilla is challenging due to inadequate bone volume resulting from alveolar ridge resorption and maxillary sinus pneumatization. This study explores the use of hyaluronic acid (HA) as a biomaterial in maxillary sinus elevation, particularly in combination with a fluid dynamic approach, as an alternative to traditional lateral approaches and granular biomaterials. **Methods**: A prospective study was conducted on 58 patients with posterior maxillary edentulism. Preoperative CBCT scans assessed residual bone height and sinus width. A minimally invasive surgical protocol utilizing a device for fluid-dynamic membrane elevation and injection of 2% cross-linked hyaluronic acid was employed, followed by simultaneous implant placement. Postoperative follow-up included a CBCT scan at 12 months to evaluate new bone height, measured mesially and distally. Implant stability was assessed using resonance frequency analysis at second-stage surgery. **Results**: A significant increase in bone height was observed at 12 months post-surgery, with an average bone gain of 7.5 mm. All 58 implants achieved primary stability, and no implant failures or signs of peri-implantitis were noted during the follow-up period. Higher bone gain was observed in wider sinuses. **Conclusions:** The fluid-dynamic transcrestal sinus floor elevation technique combined with hyaluronic acid appears to be a minimally invasive and effective method for achieving significant bone regeneration in the posterior maxilla, facilitating implant–prosthetic rehabilitation with potentially low risks and morbidity. Further large-scale studies are warranted to validate these findings across diverse clinical scenarios.

## 1. Introduction

Implant–prosthetic rehabilitation of the posterior edentulous maxilla can be challenging due to inadequate bone volume, which results from the progressive resorption of the alveolar ridge in the bucco-lingual and apico-coronal directions, as well as pneumatization of the maxillary sinus.

In 1980, Boyne and James [1] introduced a technique to promote bone regeneration in the maxilla, enabling implant–prosthetic rehabilitation in atrophic posterior alveolar ridges. This procedure involves elevating the Schneiderian membrane through a surgical lateral approach, creating a bone window in the sinus wall. Bone augmentation is then achieved by placing biomaterial—such as autologous bone particulate, bone substitutes, or a combination of both—between the alveolar crest and the lifted membrane [2].

This technique offers several advantages: the ability to regenerate substantial vertical bone height and enhanced visibility and control of membrane integrity. However, it also presents drawbacks, including high costs, a steep learning curve (especially in cases of complex sinus anatomy with septa or thin membranes), invasiveness, morbidity, and a time-consuming nature. Moreover, the procedure carries a higher risk of complications, with Schneiderian membrane perforation being the most common, along with risks of hemorrhage and infection [3,4,5,6,7].

An alternative method to increase bone volume in the posterior maxilla is the crestal approach. Originally described by Tatum [8] and later refined by Summers [9,10], this technique utilizes osteotomes to allow simultaneous implant placement with sinus lift surgery. Osteotomes, which increase in diameter and resemble the shape of tooth roots, are used to prepare the implant site. They are inserted through the edentulous alveolar crest to the sinus floor, causing a controlled fracture of the floor while preserving the integrity of the Schneiderian membrane. The graft material is then compacted, applying apical and lateral forces that elevate the membrane and create space for the implant [11,12].

Compared to the lateral approach, the crestal approach is associated with lower morbidity, but it is a highly technique-sensitive procedure. Surgeons must precisely measure the distance from the bone crest to the sinus floor to avoid membrane perforation [13,14]. Osteotomes are operated with a surgical mallet, and the percussive force can sometimes induce benign paroxysmal vertigo [15]. Fluid-dynamic transcrestal sinus floor elevation is a minimally invasive surgical technique used to augment the maxillary sinus floor for dental implant placement. Minimally invasive techniques aim to reduce patient morbidity and improve outcomes. A case report demonstrated a minimally invasive hydrostatic sinus lift using a small flap and saline solution, achieving significant sinus floor elevation and implant stability [16].

To optimize the advantages of the crestal approach, reduce complications, and simplify the learning curve, Alessandro Scarano introduced a fluid-dynamic membrane elevation technique [17]. This study aims to assess the effectiveness of this technique in patients requiring oral prosthetic implant-supported rehabilitation.

## 2. Materials and Methods

The operative technique described in this prospective study was applied to 58 patients (22 males, 36 females) aged 34–75 years (mean age 56.4 years). These healthy patients (ASA 1) were either non-smokers or light smokers (<10 cigarettes/day), with a maximum of one missing tooth (from the first premolar to the second molar) in the posterior maxilla for more than one year, presenting with insufficient bone height due to maxillary sinus pneumatization, necessitating bone augmentation and implant placement for fixed prosthetic rehabilitation. Baseline data were recorded preoperatively.

### Radiological Examination (CBCT)

Preoperative radiological assessment was conducted using Cone-Beam Computed Tomography (CBCT) to facilitate comprehensive preoperative planning. All CBCT scans were acquired using Carestream 8100 (Rochester, NY, USA). Patients were positioned according to the manufacturer’s guidelines to ensure optimal image acquisition and minimize artifacts.

The CBCT images were utilized for the precise evaluation of the following:

- Residual bone height (RBH): The vertical distance from the crest of the edentulous ridge to the floor of the maxillary sinus was measured at the intended implant sites.
- Residual bone thickness: The buccolingual width of the alveolar ridge at the intended implant sites was assessed.
- Maxillary sinus width: On cross-sectional CBCT images, the width of the maxillary sinus was measured at a point 10 mm apical to the alveolar crest. This measurement encompassed the distance between the buccal and medial cortices of the maxillary sinus.
- Sinus membrane thickness: While noted, the primary focus for linear measurements was bone dimensions.

At 12 months post-surgery, a follow-up CBCT scan was conducted. The height of the newly formed bone (final bone height) was assessed by measuring the distance mesially and distally from the highest point of the remaining crestal bone to the most apical point of the regenerated bone on cross-sectional images, with the average of these measurements recorded. The bone gain was calculated by subtracting the initial residual bone height from the final bone height. The implant protrusion length into the sinus was determined by subtracting the residual bone height from the total implant length.

The final bone height measured at 12 months post-surgery represents the primary outcome for evaluating the effectiveness of the surgical technique.

All measurements were performed using the dedicated software CS 3D Imaging Software 8 (Carestream—Rochester, NY, USA) by a calibrated examiner blinded to the surgical outcomes. Standardized protocols were followed for image analysis to ensure consistency and accuracy.

The clinical investigation was conducted at the Department of Medical Sciences of the University of Tirana, Albania, registered with Nr 276 prot. dated 4 July 2023.

## 3. Surgical Protocol

After obtaining informed consent from all patients, the surgeries were performed under local anesthesia using 4% articaine with adrenaline 1:100,000.

A palatal incision, extended intrasulcularly to the adjacent teeth, was chosen as the optimal flap design to facilitate intraoperative visibility and minimize overlap between the suture and the implant site, reducing the risk of surgical site exposure. After full-thickness flap reflection, the implant site was prepared using the M.I.S.E. kit (Sweden & Martina, Due Carrare, PD, Italy), following the manufacturer’s instructions.

Initially, a 2 mm diameter drill operating at 800 rpm was used to create a guide hole for subsequent drills. A depth stop was employed to prevent the drill from penetrating the sinus floor, leaving it intact by stopping 1 mm short of it. Following this, an intermediate drill with a 2.5 mm diameter was used, followed by a 3 mm diameter drill, both equipped with the same depth stop to gradually widen the initial hole. The implant site was then measured using the depth probe provided in the M.I.S.E. kit. A 3 mm diameter chamfered drill (C3.0) was employed, with the depth stop adjusted to the residual bone height, allowing the drill to reach the cortex of the sinus floor.

The presence of bone at the socket’s base was verified using a button probe. The subsequent steps increased the working length of the drill by 1 mm increments, ensuring gradual progression without damaging the Schneiderian membrane. The chamfered drill’s unique tip design enabled deformation of the sinus floor cortex by approximately 2–3 mm, performing a partial elevation of the sinus membrane before reaching it. Upon fracture of the sinus floor, the depth stop ensured that the drill penetrated less than 1 mm beyond the cortex, thereby preserving the Schneiderian membrane’s integrity.

Once the sinus cortex was fractured, the surgical socket was reamed at low speed (150 rpm) using the 3 mm diameter rounded drill (R3.0). For the elevation and detachment of the Schneiderian membrane, the Flusilift (Sweden & Martina) was employed as an osteotome. This device features a rounded hollow tip, allowing a syringe to be attached via a disposable plastic fitting. The kit includes a handpiece, a ring nut, and three tips of varying diameters. The Flusilift forms a seal with the surgical socket, preventing the pressurized fluid from escaping into the oral cavity and ensuring the membrane lifts properly.

The Flusilift was gently inserted into the socket using a surgical hammer until a proper seal with the bone walls was achieved, matching the residual bone height. To test the sinus membrane’s integrity, 0.5 cc of saline solution was injected into the sinus. Perforation was suspected if air was aspirated into the syringe when re-aspirating the saline. An additional integrity test was performed by observing the physiological solution mixed with blood leaking from the surgical socket upon removing the Flusilift.

**Figure 1:**
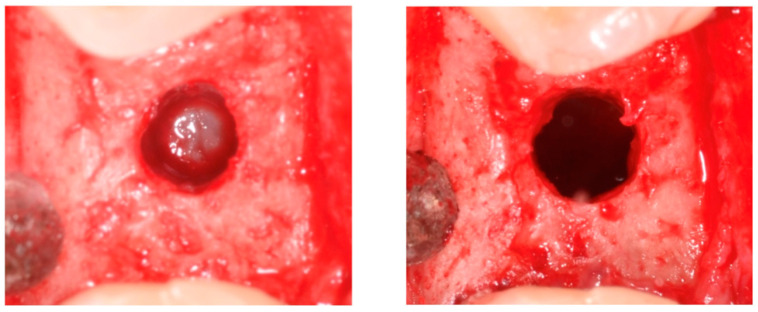
Intraoperative view of the intact sinus membrane: (**left**) demonstrating that the calibrated kit preserves the anatomical integrity of the membrane without causing perforation, (**right**) after using the Flusilift, the sinus membrane is visibly detached, creating a space that was subsequently filled with hyaluronic acid, followed by implant insertion.

**Figure 2:**
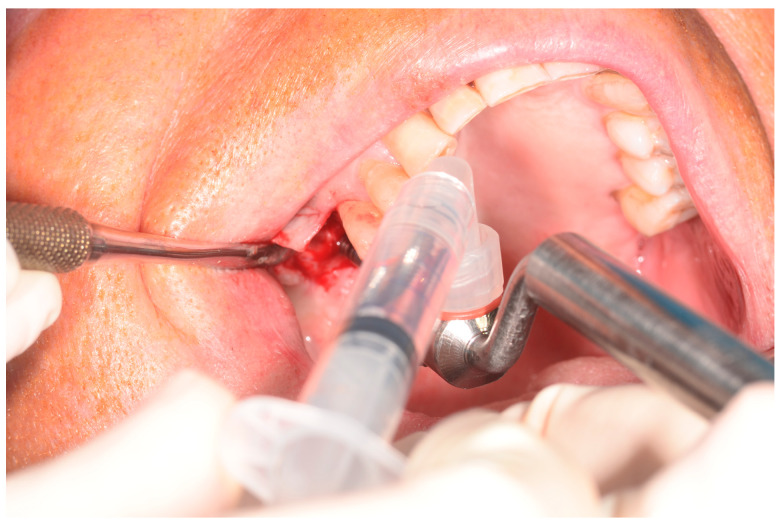
Intraoperative view of the Flusilift in action.

The Flusilift was reinserted, this time preassembled with a syringe containing 2% cross-linked, high-molecular-weight (1300 kDa) hyaluronic acid (Hyadent, Regedent, Zurich, Switzerland), and 2 cc of the biomaterial was injected. Following Pascal’s principle, the extruded fluid applied uniform pressure to the Schneiderian membrane, allowing a gentle three-dimensional detachment of the membrane in any clinical situation while minimizing the risk of perforation (Figure 1 and Figure 2).

The biomaterial, in gel form, flowed through the Flusilift and extruded from three holes near the tip—two positioned laterally 1 mm below the rounded apex and one at the apex. This distribution lifted the membrane and maintained the biomaterial in the sinus, promoting new bone formation.

In each case, a cylindrical CSR implant (Sweden & Martina) 13 mm long and with a diameter of either 4.2 or 4.5 mm was inserted. Primary stability was measured through the insertion torque, the cap screw was positioned, and the wound was closed by primary intention using a 4-0 monofilament polyamide suture.

In cases of suspected membrane perforation, the flap was closed, and the procedure was repeated after 60 days.

Postoperatively, all patients were prescribed a 6-day course of amoxicillin (1 g every 12 h), mouth rinses with 0.12% chlorhexidine for 10 days starting the day before surgery, and dexibuprofen (Seractil 400 mg) as needed.

Implants were uncovered six months after surgery, and the resonance frequency analysis was measured. Impressions were taken 20 days later, and temporary screw-retained crowns were maintained for two months with progressive loading. Final screw-retained crowns were then placed. Given that no graft biomaterial other than hyaluronic acid, which is radiolucent and rapidly resorbed, was used, it can be hypothesized that the radiopaque area around the implant represents newly formed bone. According to Albrektson, implant success was obtained in terms of implant stability, absence of peri-implant radiolucency, physiological marginal bone loss, lack of persistent pain or infection, and functional prosthetic integration [18].

## 4. Results

A total of 58 implants were inserted in the following positions: 37 in the first molar region, 5 in the second molar region, 13 in the second premolar region, and 3 in the first premolar region.

**Figure 3:**
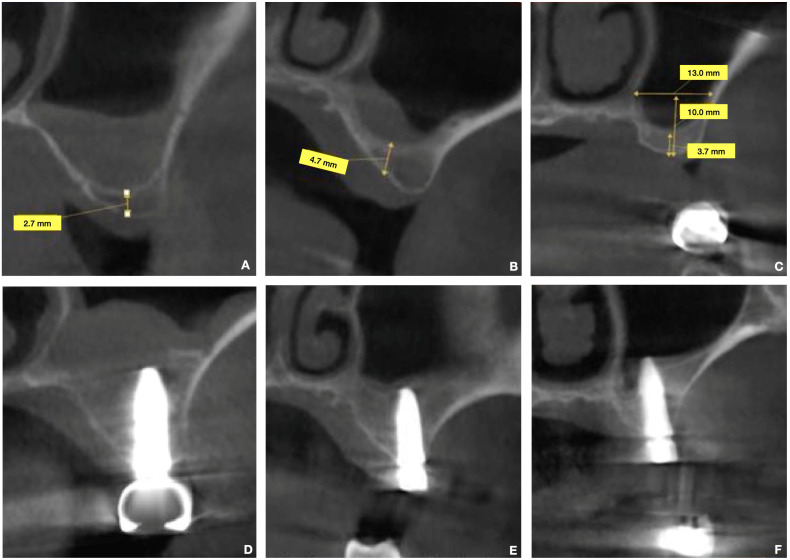
Comparison of three clinical cases. Baseline scans (**A**–**C**): The residual bone height ranges from 2.7 to 4.7 mm. CBCT scans after one-year follow-up (**D**–**F**): Show the presence of mineralized tissue surrounding the implant.

The preoperative CBCT revealed the following data: residual bone height (RBH) was 4.5 mm (SD 1.4 mm), distance from medial to lateral wall averaged 12.46 mm (SD 2.6 mm), residual bone thickness was 7.8 mm (SD 1.2 mm), and the sinus membrane measured 3.5 mm (SD 3.3 mm) (Figure 3).

During implant site preparation, no perforations occurred. All implants achieved proper primary stability, with torque values ranging from 35 to 50. At the second-stage surgery, no implant failures were observed. The Osstell values average was 71.4 (SD 2.79). All prostheses were screw-retained.

The follow-up CBCT revealed bone trabeculae around the implant. The measurement was as follows: final bone height of 12.05 mm (SD 1.2 mm) with an average bone gain of 7.5 mm (SD 1.77 mm). The implant protrusion length (IPL) into the sinus was 8.5 mm (SD 1.4 mm), with a difference between the potential regeneration and effective regeneration of 0.9 mm (SD 1.2 mm).

**Scheme 1:**
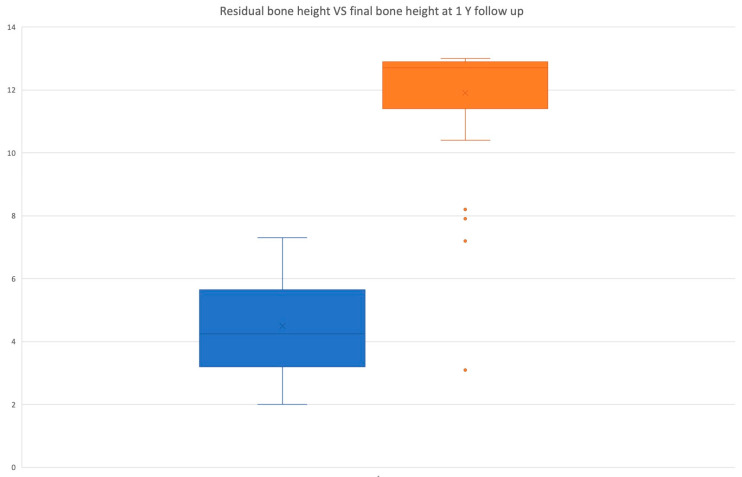
Distribution of preoperative residual bone height (blue) and final bone height measured at 1-year post-surgery (orange), represented by boxplots.

In sinuses with a medial-to-lateral wall distance greater than 12 mm, the final height was 12.24 mm (SD 1.13 mm), with a bone gain of 8.03 mm (SD 1.4 mm). In sinuses with a medial-to-lateral wall distance less than 12 mm, the final bone height was 11.78 mm (SD 1.4 mm), with a bone gain of 6.8 mm (SD 1.9 mm). No implant failures or signs of peri-implantitis were observed during follow-up (Table 1, overview of the results) (Scheme 1, overview of the differences in residual bone height and final bone height).

## 5. Discussion

The outcome of this investigation demonstrates a significant increase in bone volume following sinus lifting using the Flusilift technique combined with hyaluronic acid. This approach leverages fluid dynamic elevation of the sinus floor, which minimizes trauma and enhances the detachment of the Schneiderian membrane. The use of hyaluronic acid further supports bone regeneration by promoting faster differentiation of mesenchymal cells and facilitating mineralization. To reduce the invasiveness and morbidity associated with the Summers’ ridge augmentation technique, further developments over the years have introduced the use of controlled cutting action drills with depth stops for implant site preparation. This approach aims to avoid perforation of the sinus floor and its membrane, ensuring that only the last millimeter of the cortical bone is fractured. Following this, biomaterial is inserted into the site [17,18,19].

The crestal approach has proven to be highly effective, with high implant and prosthetic success rates, a low risk of intra-operative complications (such as Schneiderian membrane tearing and infections), and lower morbidity compared to the lateral approach. Additional advantages include shorter surgery time, reduced surgical complexity, lower risk of hemorrhage, and lower costs. However, this approach has been found to be safe and predictable only when at least 5 mm of bone is present between the atrophic alveolar crest and the maxillary sinus floor, along with a flat sinus floor and sufficient bone width for implant placement. The safety threshold for the bone height increase achievable with this technique is estimated to be 5 mm [9,10,11,20,21].

Several surgical techniques employing hydraulic pressure to elevate the Schneiderian membrane, following Pascal’s principles, are described in the literature. The introduction of a pressurized fluid tends to elevate the membrane in a uniform and controlled manner across three dimensions (apical, mesio-distal, and vestibulo-palatal), allowing for safer elevation even in significantly atrophic alveolar ridges. The detachment is more pronounced in the vestibulo-palatal and mesio-distal directions compared to conventional techniques. This procedure involves the insertion of biomaterial into the sinus along with simultaneous implant placement [17,18,19,22].

Regarding the use of granular biomaterials in both crestal and lateral approaches, numerous studies have investigated the clinical and radiographic outcomes of “graftless” procedures compared to those utilizing biomaterials. These studies have demonstrated a significant regenerative potential of the maxillary sinus even in the absence of biomaterial, with high implant success rates, comparable marginal bone loss, and new bone formation around implants after six months, following a sinus lift without biomaterial [23,24,25,26,27,28].

Autologous bone is currently regarded as the gold standard among graft materials in bone regeneration procedures. Boyne, James, and Tatum were the first to use it in maxillary sinus lift techniques [1,8].

To reduce the amount of autologous bone required and the morbidity associated with the donor site, various bone substitutes have been introduced for sinus lift procedures. Over time, numerous biomaterials have been developed as valid alternatives to autologous bone, including allografts, xenografts, and synthetic materials, which can be used alone or in combination with autologous bone. These biomaterials, in addition to being non-immunogenic, have demonstrated osteoconductive properties, although they are not entirely free from complications such as their slow resorption and management issues [28,29,30]. For example, if membrane perforation occurs during the insertion of the bone substitute, there is a risk of partial or total biomaterial loss into the sinus, potentially leading to obstruction of the osteomeatal complex and post-operative sinusitis [31]. Another potential complication is infection at the surgical site, which can spread to the graft material. Scarano et al. [32] examined bacterial proliferation within the biomaterial inserted into the maxillary sinus, demonstrating that the infection can spread from the implant surface to the graft material, necessitating complete removal of the latter in such cases.

Unlike particulate biomaterials, hyaluronic acid in gel form may mitigate these complications by significantly reducing the risk of membrane perforation during Schneiderian membrane elevation due to its solubility and fluidity. It may also minimize the risk of ostium obstruction in the event of material displacement.

Hyaluronic acid is a glycosaminoglycan, one of the main components of the connective tissue matrix, where it plays a critical role in maintaining hydration, turgidity, plasticity, and viscosity. It acts as a cementing substance and anti-shock molecule and functions as an efficient lubricant, such as in synovial fluid, preventing tissue damage from physical stress [33]. Hyaluronic acid also contributes to various cellular functions essential for tissue repair, including inflammation response, cell migration and proliferation, and extracellular matrix organization. Many biological processes mediated by hyaluronic acid are fundamental in tissue repair and wound healing, with regenerating tissues being particularly rich in this acid. In vitro studies using osteogenic stem cells have shown that hyaluronic acid significantly enhances osteogenesis [34].

When added to bone marrow stem cells in vitro, hyaluronic acid accelerates cell proliferation, increases alkaline phosphatase activity, and enhances osteocalcin gene expression. It interacts with BMP-2 to produce direct, specific cellular effects [35].

In another study, introducing hyaluronic acid into cavities experimentally induced in rat femurs produced clear signs of osteoinductive activity after just four days, contrary to control sites without hyaluronic acid [36].

Exposure to high-molecular-weight hyaluronic acid (900–2300 kDa) at appropriate dosages (0.5, 1.0, and 2.0 mg/mL) has been shown to significantly increase cell proliferation in osteoblast cultures. The study also evaluated the effect of hyaluronic acid on osteoblastic differentiation and bone formation, with positive outcomes. The authors concluded that high-molecular-weight hyaluronic acid can enhance the osteogenic and osteoconductive properties of bone grafts and substitutes due to its stimulatory effects on osteoblasts [37].

Hyaluronic acid can be categorized into linear and cross-linked (reticulated) types, depending on the treatment and composition. Cross-linking involves bonding linear hyaluronic acid molecules to create larger structures with higher molecular weight, increasing density and tissue permanence. Cross-linked hyaluronic acid is particularly suitable for tissue engineering and regenerative medicine applications due to its mechanical properties [38].

Currently, only one study in the literature evaluates the effectiveness of hyaluronic acid alone as a substitute for bone-derived biomaterials in maxillary sinus lifts in humans, in particular, the initial radiolucency, osteoinductivity, and complete resorbability of hyaluronic acid serve as valuable tools for clinicians aiming to achieve high-quality new bone formation [17]. However, numerous studies have examined its combination with biomaterials, highlighting how hyaluronic acid promotes bone regeneration by accelerating mesenchymal cell differentiation and facilitating osteoid matrix mineralization [39,40,41,42,43,44,45,46,47,48,49].

The crestal and fluid dynamic approach to maxillary sinus elevation, combined with the use of hyaluronic acid as a biomaterial, could serve as a valid alternative to the lateral approach and granular biomaterials. This technique may enable significant bone gain in the maxillary sinus, particularly in patients with residual bone height of less than 4 mm. The extent of this bone gain is influenced by the degree of implant protrusion into the sinus, which creates a “curtain effect”, as previously demonstrated in the literature [50].

Stacchi demonstrated that the conventional crestal approach is less predictable in wide sinuses, with a six-month bone gain of only 3% in sinuses wider than 15 mm at the implant site [51]. In our study, we assessed the enhanced sinus bone gain (ESBG) at 12 months using the fluid dynamic technique with hyaluronic acid, correlating it with sinus width at the implant site. Unlike the traditional osteotome technique, which provides limited membrane detachment, the fluid dynamic technique enables more extensive detachment, reaching the vestibular and palatal walls of larger sinuses. This allows the graft to have greater contact with the bone, extending to the most apical peaks.

Notably, the high thickness of Schneider’s membrane did not appear to hinder the bone regeneration process. A significant difference was also observed in the shape and distribution of the graft between the osteotome technique and the fluid dynamic approach. In the osteotome technique, the biomaterial is pushed only in the corono-apical direction, initially detaching the membrane modestly before stretching it to the point where rupture occurs. In contrast, the fluid dynamic technique, based on Pascal’s principle, allows for uniform three-dimensional membrane detachment without the constraints of stretching, enabling elevations of up to 18–20 mm in all spatial dimensions. This results in a dome-shaped distribution of the biomaterial around the implant within the newly formed space [17,18].

The decision to use hyaluronic acid alone, without heterologous bone granules, was based on the observation that the natural blood clot within the maxillary sinus already possesses maximum regenerative potential. It supports bone healing comparably to BMP-2, remains incompressible, and, being fluid, does not cause complications in the event of membrane perforation [52].

Hyaluronic acid shows promise in targeted drug delivery due to its biocompatibility and receptor interactions. It is crucial for tissue engineering and regenerative medicine, providing scaffolds for cell growth [53,54,55]. HA aids wound healing by promoting cell activity and is explored for advanced ocular and joint therapies. Its dental applications include guided tissue regeneration for periodontal defects, cyst cavities, extraction, and endodontics [56,57,58,59,60].

Additionally, the absence of pain, edema, hematoma, and bleeding may be attributed to the minimally invasive nature of the surgical procedure. However, the main limitation of this technique is the necessity of achieving primary implant stability and ensuring a proper seal of the surgical socket, as the implant must always be placed simultaneously with the procedure.

Several limitations warrant consideration when interpreting the findings of this study. Firstly, the presence of metal artifacts from the prosthetic components could have potentially influenced the accuracy of CBCT measurements. Secondly, the absence of a control group limits our ability to definitively attribute the observed bone regeneration solely to the described fluid-dynamic technique and hyaluronic acid. Future studies incorporating a control group undergoing a different treatment modality or no treatment would provide a stronger basis for comparison. Finally, the lack of a formal sample size calculation prior to this study may have impacted the statistical power to detect smaller, yet potentially clinically significant, differences.

## 6. Conclusions

The goal of regenerative bone surgery is to achieve high-quality bone regeneration using less invasive techniques and with a reduced risk of complications. The fluid-dynamic technique described in this case series, combined with the use of hyaluronic acid as a biomaterial, achieved a high volume of regenerated bone with low risks, low cost, and low morbidity.

Further large-scale studies are needed to thoroughly investigate the characteristics of this surgical procedure across various clinical scenarios, especially in cases involving large and fully pneumatized maxillary sinuses.

## Figures and Tables

**Table 1:** Results.

Category	Value
Total Implants	58
Residual Bone Height (RBH)	4.5 ± 1.4 mm
Medial-Lateral Wall Distance	12.46 ± 2.6 mm
Residual Bone Thickness	7.8 ± 1.2 mm
Sinus Membrane Thickness	3.5 ± 3.3 mm
Torque (Primary Stability)	35–50 Ncm
Osstell Value (ISQ)	71.4 ± 2.79
Final Bone Height	12.05 ± 1.2 mm
Bone Gain	7.5 ± 1.77 mm
Implant Protrusion Length (IPL)	8.5 ± 1.4 mm
Regeneration Difference	0.9 ± 1.2 mm

## Data Availability

The original contributions presented in this study are included in the article. Further inquiries can be directed to the corresponding author.
